# Functional metagenomic libraries generated from anthropogenically impacted environments reveal importance of metabolic genes in biocide and antibiotic resistance

**DOI:** 10.1016/j.crmicr.2023.100184

**Published:** 2023-02-26

**Authors:** Aimee K. Murray, Lihong Zhang, Jason Snape, William H. Gaze

**Affiliations:** a European Centre for Environment and Human Health, University of Exeter Medical School, Environment and Sustainability Institute, Penryn Campus, Cornwall TR10 9FE, United Kingdom; b AstraZeneca Global Environment, Alderly Park, Macclesfield, United Kingdom

**Keywords:** Antimicrobial resistance, Biocide, Functional metagenomics

## Abstract

Anthropogenic activities result in the release of antimicrobial resistant bacteria and a cocktail of antimicrobial compounds into the environment that may directly select or indirectly co-select for antimicrobial resistance (AMR). Many studies use metagenome sequencing or qPCR-based approaches to study the environmental resistome but these methods are limited by *a priori* knowledge. In this study, a functional metagenomic approach was used to explore biocide resistance mechanisms in two contaminated environments and a pristine site, and to identify whether potentially novel genes conferring biocide resistance also conferred resistance or reduced susceptibility to antibiotics. Resistance was predominately mediated through novel mechanisms exclusive of the well-known *qac* efflux genes. UDP-galactose 4-epimerase (*galE*) –like genes were identified in both contaminated environments and were shown to confer cross-resistance to biocides and clinically important antibiotics for the first time (to our knowledge), compared to knockout mutants. *GalE* -like genes were also co-located with transposons, suggesting mobilisation potential. These results show that housekeeping genes may play a significant yet underappreciated role in AMR in environmental microbiomes.

## Highlights

• Functional libraries from three environments were screened for biocide resistance.
• Biocide resistance was common in textile effluent remediating soil and sewage cake.
• Putative resistance genes were characterised.
• *GalE* like genes were most common and conferred reduced antibiotic susceptibility.
• *GalE* like genes were potentially mobilizable.

## Graphical abstract

**Figure:**
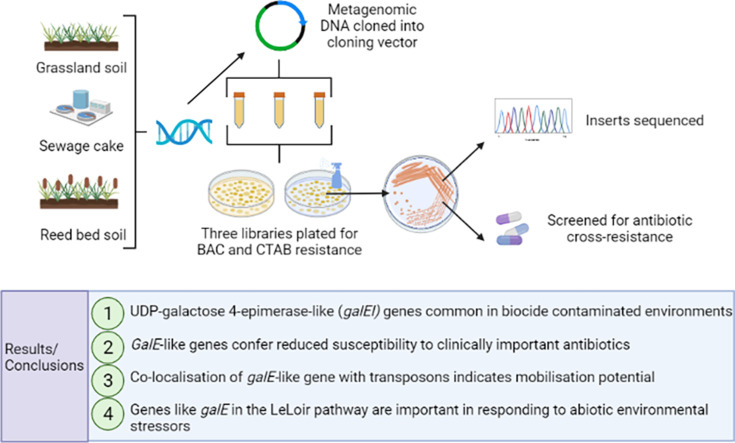

## Introduction

The extensive use and misuse of antimicrobials in modern medicine, farming and aquaculture (Laxminarayan et al., 2013; Livermore 2009) has increased the emergence and spread of antimicrobial resistance (AMR). Quaternary ammonium compounds (QACs) are biocides, antimicrobial compounds used not as therapeutic agents, but as surfactants, detergents and disinfectants (Oh et al., 2013) and as preservatives in personal care products (Buffet-Bataillon et al., 2012). Their use precedes that of antibiotics (Gillings et al., 2009) and they are estimated to be produced in quantities several orders of magnitude greater than antibiotics (Calero-Caceres et al., 2014). Due to their wide use and poor biodegradability (Buffet-Bataillon et al., 2012; Oh et al., 2013), they may exert a greater selective pressure on AMR in the environment than antibiotics, which are generally found at very low concentrations (aus der Beek et al. 2016).

Selection for AMR can occur following direct exposure to antibiotics but also indirectly, through exposure to other antimicrobials like biocides via co-selection. Co-selection can be defined as selection for a resistance gene when 1) it confers resistance to more than one antibiotic/compound (cross-resistance); or 2) when it is genetically linked to or on the same mobile genetic element as another gene under positive selection (co-resistance) (Baker-Austin et al., 2006). Co-selection of biocide and antibiotic resistance via co-resistance has been shown to occur under laboratory conditions (Tandukar et al., 2013) and there is evidence for co-resistance of biocide and antibiotic resistance genes in the environment (Drudge et al., 2012; Gaze et al., 2005; Gaze et al., 2011). Still, more work is required to understand the role of co-selection in the generation, maintenance and spread of AMR (Andersson and Hughes 2014).

Studies on the environmental resistome are now largely through culture-independent methods such as qPCR and metagenomics; however, this limits the findings to rediscovery of previously characterised genes or those with significant similarity. It is important to continue to functionally characterise novel resistance genes, to improve the databases upon which culture-independent methods rely. Furthermore, a recent experimental evolution study has shown mutations that confer antibiotic resistance can frequently occur in noncanonical genes (Lopatkin et al., 2021), limiting the comprehensiveness of studies requiring *a priori* knowledge even further. Functional metagenomic approaches may also be useful for identifying novel AMR genes in the environment that may become clinically problematic in the future. For example, the extended spectrum β-lactamase *bla*_CTX−M_ genes originated from the chromosome of environmental *Kluyvera* species (Humeniuk et al., 2002; Olson et al., 2005). Following mobilisation events, these genes have achieved pandemic distribution worldwide in diverse bacterial pathogen backgrounds (Cantón et al., 2012).

A previous study (Zhang et al., 2019) employed a functional metagenomic approach to detect genes encoding resistance to extended spectrum β-lactams and carbapenems in sewage sludge, sludge amended soil, QAC impacted reed bed soil and less impacted grassland soil. Six novel ESBL and carbapenemase genes were identified in the metagenomic libraries generated from these samples, with varying degrees of homology to clinically important β-lactamase genes. The sewage sludge and the QAC contaminated soil appeared to enrich for ESBL genes which were not detected in long term curated grassland soils.

In this study, we aimed to test the hypothesis that industrial waste and sewage sludge contribute to environmental reservoirs of antimicrobial resistance through introduction or enrichment of biocide resistant bacteria with resistance mechanisms that confer cross-resistance to antibiotics. Functional metagenomic libraries (Zhang et al., 2019) were screened for resistance to two biocides: benzalkonium chloride (BKC) and cetyltrimethylammonium bromide (CTAB). Co-resistance to QACs and antibiotics was phenotypically screened, and putative QAC resistance genes were functionally confirmed and screened for cross-resistance to antibiotics. This study confirms the importance of functional metagenomics in environmental resistome characterisation, as a novel, noncanonical clade of resistance genes was found that conferred cross-resistance to biocides and antibiotics in both anthropogenically impacted sites.

## Materials & methods

### Sampling sites and metagenomic library construction

The metagenomic library construction involved cloning total metagenomic DNA extracted from environmental samples into *Escherichia coli*. The *E. coli* contain different fragments of metagenomic DNA, which may include entire genes and their natural promoters. If the cloned DNA fragments contain biocide resistance genes or antibiotic resistance genes, they will confer new resistance phenotypes to the host. Therefore, resistant clones can be identified by plating the cloned *E. coli* onto biocide or antibiotic containing selective media.

The metagenomic libraries used were generated in a previous study (see (Zhang et al., 2019) for full methods) that focused on β-lactamase discovery. In brief, the samples/sites chosen were: fully digested, dehydrated and limed sewage cake (SC) collected from a West Midland wastewater treatment plant (WWTP), reed bed soil (RB) amended with effluent from a textile mill with high usage of QACs (Gaze et al., 2005) and grass land (GL) soils. The GL samples were taken from the Rothamsted Park experimental grassland plots, which have been protected from anthropogenic impact for the past 150 years (SILVERTOWN et al., 2006). The metagenomic libraries constructed using the cloning plasmid pCF430 (Newman and Fuqua 1999) and the cloned DNA were transformed into *E. coli* TransforMax EC100 competent cells (Epicentre).

The total number of clones was estimated by plating out a small fraction of the libraries on selective LB plates with 10 µg/ml tetracycline (TET). Then, 30 clones from each library were randomly chosen for plasmid extraction and the DNA inserts were excised with BamH1 enzymes. The sizes of each insert were estimated on agarose gel. The percentage of positive clones is the number of positive clones (with inserts) divided by the total number of clones tested. The average insert size of the positive clones (the clones with inserts) equals the subtotal insert sizes of positive clones divided by the number of positive clones tested. The coverage of each library equals the number of clones multiplied by the average insert size of the positive clones and multiplied by the percentage of positive clones (Table S1). Coverage is used to represent the total DNA (in bp) that was cloned in the original metagenomic libraries. Because the original metagenomic libraries were first whole-genome amplified within the original bacterial hosts, the amplified libraries contain multiple copies of the original genomes. The one-time coverage takes this into account, and is therefore normalised to the original libraries’ DNA length. Since different libraries were amplified to different levels, 5 × 100 µl contains one-time coverage for the RB and GL libraries while 50 µl contains one-time coverage for the SC library (i.e., RB and GL were amplified 100x more than SC).

### Culturing and identifying unique inserts

All culturing (liquid and in agar, both LB) contained TET 12.5 μg/ml (Sigma Aldrich, UK) for vector maintenance. Incubation was stationary or shaking (150–180 rpm) at 35 - 37 °C overnight.

Libraries were screened by plating on five LB agar plates containing MIC (of host with empty vector) concentrations of BKC (Sigma Aldrich, UK) and CTAB (Sigma Aldrich, UK) at 16 μg/ml and 32 μg/ml respectively, with 100μl of RB, GL or SC libraries, which equates to one coverage per library across the five plates. Chi-squared tests were used to determine if numbers of resistant colonies differed between libraries or QAC.

Given CTAB and BKC are both QACs and are therefore likely to have similar resistance mechanisms, we focused on BKC resistant clones for further characterisation. We then did a high-level screen for cross-resistance in CTAB resistant clones as we would not be characterising these further (see MIC/Co-selection testing section). A high-level screen was not performed for BKC resistant clones as we planned to investigate these in more depth.

Plasmid DNA extraction was performed on a random selection of 24 BKC resistant clones from both the RB and SC library alongside the single BKC resistant clone isolated from GL with the GeneJet MiniPrep Kit (ThermoScientific, UK), according to instructions for low plasmid copy number. Restriction digest with EcoRI and *Bam*HI FastDigest Green (ThermoScientific, UK) identified unique inserts via visualisation on a 0.8% agarose gel.

### Gene knock out and sequencing

Unique vectors underwent transposon mutagenesis using the EpiCentre Kan-25 EZ Kit, according to instructions (Epicentre, UK). Mutagenized vectors were electroporated into electrocompetent EC100 cells (Epicentre, UK). Successfully shocked cells (pulse time >4.0 ms) were recovered in 500μl SOC media and incubated for 60 - 90minutes. Cells (100μl) were diluted to 10^−3^ in LB broth and spread on plates containing TET only, to assess transformation success. The remainder of cells were spread in 100μl aliquots onto LB agar plates containing kanamycin (KAN) at 50 μg/ml (Sigma Aldrich, UK), to select for clones with successful transposon insertion and TET. ClonTech (UK) chemically competent (Stellar™) cells were also used for transformation according to manufacturing conditions.

Following incubation overnight, clones were randomly selected to be functionally screened for resistance gene knockouts. Mutants were spotted onto LB agar plates containing KAN (50 μg/ml) and TET and onto LB agar plates containing KAN (50 μg/ml), TET and BKC at 16 μg/ml, 24 μg/ml or 32 μg/ml (to assess the extent to which resistance was lost). Over 120 mutants were screened per mutagenesis reaction. Clones unable to grow on one or more of the BKC plates were picked from the plates without BKC and grown overnight in 10 ml (due to low plasmid copy number) LB Broth for plasmid extraction as described above.

Restriction digest (as above) determined unique mutants for the transposon reactions with mixed vectors, and these along with the single vector transposon reaction knock outs were sent for sequencing (GATC Biotech) using the forward and reverse primers provided in the EpiCentre Kan-25-EZ Kit. For sequence analysis, the transposon sequence was removed and forward and reverse sequences were combined to give the knock out gene sequence using MEGA5.2 (Tamura et al., 2011). GenBank Open Reading Frame (ORF) finder was used to identify potential ORFs which then underwent BLASTp. Entire inserts were also sequenced to search for co-resistance, by primer (IDTDNA, UK) walking using plasmid DNA extracted as above (sequencing performed by Macrogen Europe, Netherlands).

### Phylogenetic analysis

Phylogenetic analyses were performed in MEGA 6.0 (Tamura et al., 2013). *GalE*-like sequences identified in this study were aligned with a *galE* gene from GenBank (Accession NC004663.1). A maximum likelihood tree with 500 bootstrap replicates based on the trimmed sequences was used to identify 7 distinct groups of *galE*-like genes. One *galE*-like ORF from 3 of these subgroups were selected to be cloned into an expression vector, to screen for cross-resistance.

### *Cloning* galE *-like ORFs, and expression*

One ORF (R11) was successfully amplified using Q5 enzyme (New England Biolabs, UK). The other two ORFs (R161 and R241) were amplified using HotStart OneTaq (New England Biolabs, UK) enzyme. Products were run on a 0.8% agarose gel to verify successful amplification of a single specific band. All reactions were cleaned up using the NucleoSpin Clean and Concentrate kit (Mackery-Nagel, UK), according to manufacturer's instructions. PCR products were also sequenced before cloning (Macrogen Europe, Netherlands) to verify high fidelity amplification.

R11 was successfully cloned into the pET101 vector using the Champion pET Directional Expression kit (InVitrogen, UK) according to the manufacturer's instructions, and was electroporated as above. The other two ORFs (R161 and R241) could not be successfully cloned using the pET101 kit, so they were cloned using a published protocol called ‘Hot Fusion’ (Fu et al., 2014). Colony PCR (conditions as above) performed with the T7 forward and reverse primers confirmed the ORF had been successfully cloned into the vector. The vector was also sequenced (Macrogen Europe, Netherlands) using primers provided in the Champion Directional Expression kit (T7 forward and reverse) to confirm the insert was in the correct orientation.

All *galE*-like ORFs and the positive control expression vector were chemically transformed into the OneShot (InVitrogen, UK) expression cells according to instructions. Transformed cells were incubated for 30 min at 37 °C at 180 rpm, then added to 10 ml LB broth with ampicillin (AMP) (50 mg/L) and incubated overnight. This culture was used for the initial disc diffusion assays. Freezer stocks were made as described above and cultured from two consecutive single colony and overnight cultures before being used for E-test assays.

### MIC/Co-selection testing

MIC testing of the resistant clones was performed using Iso-sensitest agar, using the two 5 μl spot method of overnight culture diluted to OD 600 nm 0.25 (Andrews, 2001). EC100 pCF430 (host with empty vector) was used as a negative control. This was performed to determine the BKC MIC for all the resistant isolates from the metagenomic libraries (i.e., the full insert, containing *galE*-like ORFs and other DNA), as well as *E. coli* which had only the subcloned *galE*-like ORFs.

To determine the numbers of clones resistant to BKC or CTAB, first, the total numbers across all plates were corrected for library coverage. Coverages for RB, SC and GL were 302 μl, 7.42 μl and 306 μl, respectively. Library coverage was determined by dividing the amount of library plated across the 5 plates (500 μl for RB and GL, and 50 μl for SC) by the volume of a single coverage. Total numbers of resistant clones for each library and each treatment (BKC or CTAB) were divided by this library coverage and rounded to the nearest whole value.

As most characterisation focused on BKC inserts, we also performed a high level screen for evidence of co-selection of CTAB and antibiotic resistance. The entire RB and SC libraries were also screened on AMP and trimethoprim (TRMP) to investigate possible co-resistance of CTAB and antibiotic resistance genes in the insert DNA. Approximately similar numbers of clones (∼ 200) were isolated by plating on one plate containing only TET, and another containing CTAB (32 mg/L) and TET. These were replica plated onto agar containing AMP or TRMP at the minimum inhibitory concentration (MIC) of the host with empty vector (4 and 1.5 mg/L, respectively), approximately 1.5x this MIC (7 mg/L and 2.25 mg/L, respectively) and at clinical breakpoint concentrations for *Enterobacteriaceae* (8 and 4 mg/L, respectively (EUCAST 2014)), to reflect the *E. coli* host background.

Original BKC resistant inserts and cloned *galE*-like ORFs were tested for increased antibiotic resistance compared to the empty vector control (pCF430 for original inserts, or pET101 for *galE*-like ORFs) with disc diffusion assays (Fisher-Scientific, UK). Antibiotic susceptibility disc diffusion assays were performed for Doxycycline hydrochloride (30 μg) and Minocyline (30 μg) for the *galE*-like ORFs only (as all other vectors contained TET resistance); and AMP (10 μg) for the original inserts only (as *galE*-like ORF containing vectors contained an AMP resistance gene). Both *galE*-like ORFs and original inserts were also screened on TRMP (2.5 μg), Cefotaxime (‘TAX’ 5 μg), Imipenem (‘IMP’ 10 μg), Colistin (‘COL’ 10 µg) and Sulfamethoxazole (‘SMX’ 25 μg). Testing was performed according to EUCAST standards (Matuschek et al., 2014). Negative control expression plates without IPTG were included, as well as the positive control expression vector plated as above on plates also containing X-Gal (to allow for visual confirmation of expression through blue/white screening). Disc diffusion assays were performed in triplicate for all three expression vectors and for the expression vector control. Inhibition zone sizes were compared using an ANOVA and Tukey post-hoc test for significance. E-tests (Biomerieux, UK) for SMX, TRMP and SMX / TRMP were also performed on *galE*-like genes with appropriate controls following EUCAST standards, using Muller Hinton (MH) agar supplemented as above. The graphical abstract was created with Biorender.com.

## Results

### Evidence of co-resistance

Libraries from anthropogenically impacted matrices, including a constructed reed bed receiving biocides and detergent rich waste from a textile mill (RB) and sewage cake (SC) that has been exposed to biocide and detergent rich effluent had significantly higher numbers of resistant clones per Gb of library compared to the grassland soil (GL, all *p* <0.001, Chi-sq test). Numbers of clones resistant to BKC or CTAB per Gb of library were significantly different in the RB library (*p* <0.001, Chi-sq test) but there were no significant differences between treatments in the SC or GL libraries (see Table 1).

**Table 1:** Numbers of BKC or CTAB resistant colonies from each of the three metagenomics libraries from the initial screen, and corrected for per Gb of library, rounded to the nearest whole number. The library coverages are 0.63, 1.59 and 1.53 Gb for RB, SC and GL, respectively. * = significantly different to GL, *p* < 0.05, Chi-Sq Test.

**Library**	**No. BKC resistant**	**No. CTAB resistant**	**No. BKC resistant per Gb library**	**No. CTAB resistant per Gb library**
**RB**	397	648	151*	260*
**SC**	160	240	38*	57*
**GL**	1	5	1	5

An initial screen for co-selection of antibiotic resistance found greater than 80% of all CTAB resistant clones in the RB and SC libraries also had reduced susceptibility to both TRMP and AMP (1.5 × 1.75x the empty vector control MIC, respectively). However, clinically relevant resistance was extremely rare (1 CTAB resistant clone was also clinically resistant to TRMP, i.e. had an MIC > 4 mg/L, the EUCAST defined clinical breakpoint concentration (EUCAST 2014)).

To identify the genes responsible for BKC resistance (i.e., BKC MIC >16 mg/L), 8, 6, and 1 unique BKC resistant clones were identified for the RB, SC and GL libraries, respectively via restriction digestion. Full sequencing of the ‘unique’ inserts identified five as identical to one of the other inserts (based on ∼2000 bp in both forward and reverse having 100% homology), so sequencing of these was discontinued. The full sequences for the remaining unique inserts were searched for genes that could potentially confer AMR, through ORF Finder and BLASTp searches.

The full list of ORFs, their predicted function and identity are in Table S1. Three inserts contained ORFs with varying similarity to known antibiotic resistance genes. S4 contained a tetracycline resistance MFS efflux pump (100% ID) and multidrug resistance protein mdtB (42% ID); and R3 contained a penicillin binding protein (30% ID). S4 also contained part of a transposase (100% ID but only 42% coverage), suggesting recent mobilisation of a tetracycline MFS efflux pump and the potential for further transfer. Insert R10 also contained a transposase, suggesting co-localised genes such as the ABC transporter protein and *galE*-like gene (see below) also within this insert are potentially mobilisable. In terms of biocide resistance, the only notable ORF was found in insert R24, which contained a sulfatase (up to 96% ID).

Other key ORFs identified could be grouped by predicted resistance mechanism strategy. For membrane/transporter/efflux pumps, there were hits for ABC transporters, MFS transporters, a FIST-domain containing protein (involved in transport and binding of small ligands) and membrane proteins. For preventing cell lysis/penetration, there were hits for a predicted capsular polysaccharide biosynthesis protein and cell wall/membrane synthesis proteins including the *galE*-like ORFs and a UDP-galactopyranose-mutase. To combat cell damage, there were hits for an oxidoreductase and a divalent ion tolerance protein. In one of the inserts, there was high sequence identity and coverage for 16S rRNA and several other genes (including an ABC transporter) from *Pseudomonas veronii*.

### Evidence of cross-resistance

For the transposon mutagenesis knockouts, the ORF name, sequence similarity and number of mutant clones containing knocked out ORFs for both RB and SC libraries are shown in Table 2. G*alE*-like genes were present in the majority of knockouts; these have been shown in previous studies to confer resistance to tetracyclines (Kazimierczak et al., 2009), and *galE* mutants have been shown to exhibit increased susceptibility to clinically important antibiotics such as TAX, IMP, and vancomycin (Nakao et al., 2006; Nayak et al., 2006). Alignment of *galE-*like ORFs from this study with the ORFs identified previously (Kazimierczak et al., 2009) found limited sequence similarity (Table S3).

**Table 2:** Predicted ORFs, amino acid identity, and the numbers of knock outs containing this ORF for transposon knock-out mutants.

No. knock outs	ORF name	% amino acid identity
10	UDP-galactose-4-epimerase	66 - 99
4	‘Hypothetical protein’	33 - 98
2	Drug/metabolite transporter permease/PecM-like protein	96 - 99
2	KAP family P-loop domain protein	87 - 98
2	Putative MFS transporter protein	36 - 40
1	Multidrug transporter/transporter permease/PecM-like protein/integral membrane protein	96 - 98
1	Putatative membrane-associated metal-dependant hydrolase	78
1	Phage T7 exclusion protein	78
1	Oxidoreductase	69
1	Putative polysaccharide transport system component signal protein	46
1	Quinone/putative oxidoreductase	34

**Fig. 1:**
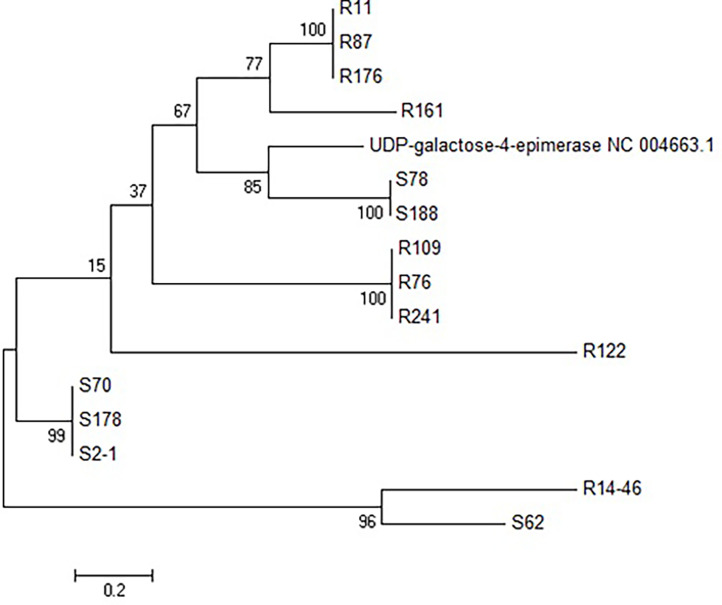
Maximum likelihood tree of the UDP-galactose 4-epimerases identified in this study, with a reference strain from GenBank (Accession NC 004663.1). Bootstrap values based on 500 bootstrap replicates. Sequences beginning with R are from the RB library, and with S from the SC library.

**Fig. 2:**
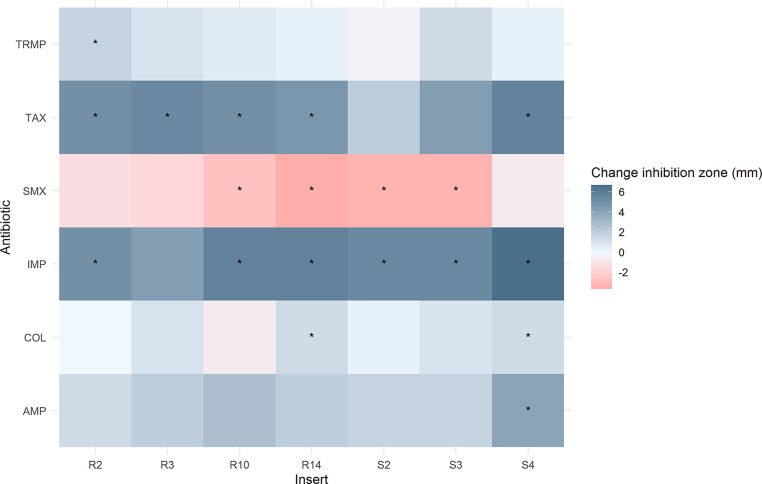
Change in average halo size following disc diffusion assays for the original benzalkonium chloride resistant clones (R – Reed Bed, S – Sewage Cake) compared to the empty vector control (i.e., average halo size of the insert carrying isolate, minus the average halo size of the empty vector control, so a negative value indicates increased resistance). ‘*‘ indicates significantly different (*p* < 0.05, ANOVA with post-hoc Tukey test).

**Fig. 3:**
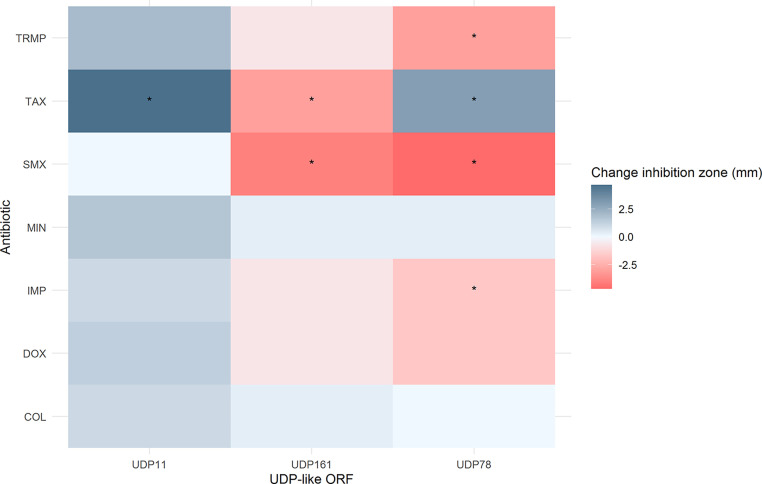
Change in average halo size following disc diffusion assays for the *gale* -like genes compared to the empty vector control (i.e., *galE* -like gene containing subclones isolate, minus the empty vector control average halo size, so a negative value indicates increased resistance). ‘*’ indicates significantly different (*p* < 0.05, ANOVA with post-hoc Tukey test).

A phylogenetic analysis (Fig. 1) was conducted to visualise the similarity between knock out *galE*-like genes. Three ORFs from three different subgroups resulting from this analysis were sub-cloned and antibiotic disc diffusion assays were conducted to determine the antibiotic susceptibility profiles of the original BKC resistant clones and these three sub-cloned *galE*-like ORFs. For the original BKC resistant clones, possession of the insert significantly increased susceptibility to IMP and, for S4, to AMP (all *p* < 0.05, ANOVA with Tukey post-hoc test, Fig. 2). For the *galE*-like ORFs (Fig. 3), R11 and S78 showed significant increases in susceptibility to TAX (*p* < 0.01 and *p* < 0.05, respectively), whereas R161 showed a significant decrease (*p* < 0.05). All three showed significant decreased susceptibility to SMX (all *p* < 0.01), and S78 also showed a significant decrease in susceptibility to IMP (*p* < 0.05) and TRMP (*p* < 0.05).

To verify these results and determine the difference in MIC, MIC strip (E test) assays were performed (Table 3). Only R11 showed an increase in TRMP MIC compared to the empty vector control. For SMX, the increase in MIC was more pronounced, with the MIC of R11 and S78 tripling that of the control. TRMP/SMX combination MICs were also determined as this is a common therapeutic combination (Sirtori et al., 2010). The average TRMP/SMX MICs increased in all vectors containing *galE-*like ORFs compared to the control. Cloned *galE*-like ORFs also showed elevated levels of BKC resistance compared to the expression vector control (from > 2 and < 4 mg/L; to > 4 mg/L and 〈 6 mg/L for R11 and R161 or 〉 6 and < 8 mg/L for S78).

**Table 3:** E test MIC results for cloned UDP-like ORFs. TRMP = Trimethoprim, SMX = Sulfamethoxazole, TRMP / SMX = Trimethoprim / Sulfamethoxazole combination strip. '^' represents increase in MIC, '-' represents no difference in MIC, '˅' = decrease in MIC (all compared to empty vector control pET101).

Antibiotic	Empty vector control pET101 MIC (µg/ml)	R11 MIC (µg/ml)	R161 MIC (µg/ml)	S78 MIC (µg/ml)
TRMP	0.19	0.25 ^	0.19 -	0.125 ˅
SMX	2	6 ^	2 -	6 ^
TRMP / SMX	0.019	0.0255 ^	0.0275 ^	0.032 ^

## Discussion

### Co-resistance

BKC and CTAB are both common QACs with applications as preservatives in pharmaceuticals, personal care products and in household cleaning products and detergents (Buffet-Bataillon et al., 2012). As both the RB and SC environments were expected to be exposed to QACs, it is unsurprising that higher numbers of QAC resistant clones were isolated from the RB and SC environments, compared to the GL environment. However, this could also reflect the different compositions of the microbial communities that exist in these environments.

Reduced susceptibility to AMP and TRMP was common in CTAB resistant clones isolated from both impacted libraries. However, resistance to either antibiotic was greater in the RB library, which was likely exposed to higher QAC concentrations. Though clinical resistance was extremely rare, this does not render the findings of this study clinically insignificant. As the expression of the inserts was controlled by their natural promoters, it is possible higher levels of resistance may be achieved under different expression levels, suggesting these mechanisms may form part of the ‘proto-resistome’ (Perry et al., 2014). Genetic context has been shown to greatly influence MIC previously (Amos et al., 2014), so mobilisation into a new genetic context (which with some of the inserts would be possible, due to the presence of a transposase) could result in higher levels of expression; or resistance could be achieved through multiple copies and a gene dosing effect (Martinez and Baquero 2000). Host context may also play a role in the level of resistance conferred.

For some unique biocide resistant clones, not even a single knockout mutant could not be obtained. It is possible that these inserts contained more than one gene conferring resistance to BKC as complete loss of phenotypic resistance was not observed. Therefore, these inserts were sequenced fully by primer walking. This simultaneously allowed for screening for co-resistance candidates, i.e., QAC and antibiotic resistance genes clustering within the same insert. There was some evidence of this, including a potentially recently mobilised tetracycline resistance MFS efflux pump (100% ID) co-located with a transposase, and a sulfatase previously shown to be able to degrade anionic surfactants (Jovcic et al., 2010).

Many ORFs had varying levels of similarity to efflux pumps (MFS/ABC transporter), genes involved in cell wall/polysaccharide capsule synthesis; or genes involved in reducing reactive oxygen species (oxidoreductase, nitrilase). These genes would need further characterisation to confirm a functional role in either biocide or antibiotic resistance. It was difficult to discern the host species from insert sequences, as most had a mixture of different host species within individual inserts (Supplementary Table 1). However, it was clear one insert was from *P. veronii,* which has been shown previously to have potential bioremediation capabilities (Nam et al., 2003; Onaca et al., 2007), suggesting anthropogenically impacted environments may favour growth of bioremediating species.

### Cross-resistance

Recent research has highlighted mutations in canonical AMR genes do not fully represent the dearth of mutations that can confer AMR. Lopatkin et al. (2021) found that mutations occurred at similar or even higher levels in metabolism genes than in canonical AMR genes in experimentally evolved *E. coli;* often, these mutations conferred resistance to more than one antibiotic*.* Our study supports this finding and suggests this may be a common strategy adopted by environmental bacteria facing a variety of abiotic stressors. Of all the putative genes identified in this study conferring biocide resistance, only the genes encoding a hydrolase (Su et al., 2014) and *galE*-like genes (Kazimierczak et al., 2009; Nakao et al., 2006; Nayak et al., 2006) have been reported to also confer antibiotic resistance. *GalE* genes can also confer resistance to QACs (Tansirichaiya et al., 2017) and the antifungal drug ciclopirox (Carlson-Banning et al., 2013), as well as increase salt tolerance (Culligan et al., 2012). However, to our knowledge, this is the first time that cross-resistance to QACs and antibiotics has been demonstrated for several distinct *galE-*like genes isolated from sewage and soil microbiomes.

*GalE* encodes UDP-galactose 4-epimerase, which converts UDP-galactose into UDP-glucose, as the final step in the Leloir galactose metabolism pathway (Chai et al., 2012). A previous functional metagenomic study in soil also found low level carbapenem resistance could be conferred by putative galactose-1-phosphate uridyltransferase (galT) enzymes (Djenadi et al., 2018), which is another key enzyme in the Leloir pathway (Chai et al., 2012). Multiple studies have demonstrated the importance of *galE* in biofilm and lipopolysaccharide formation for a variety of species (Carlson-Banning et al., 2013; Chai et al., 2012; Fry et al., 2000; Jennings et al., 1994; Nakao et al., 2006; Niou et al., 2009; Robertson et al., 1993; Tansirichaiya et al., 2017; Zou et al., 2013), suggesting reduced cell penetration is the likely resistance mechanism. It is unknown whether the genes isolated in this study still form part of this metabolic pathway within their hosts, if they are duplicated, mutated genes, or if the primary function is AMR. It is important to note that unlike the original inserts (where expression was controlled by natural promoters), the expression of the *galE*-like ORFs were under control of the non-native, IPTG-inducible T7 promoter. This was necessary in order to confirm protein function, but means expression in the natural environment may differ and this could affect the levels of susceptibility conferred by these *galE*-like ORFs *in situ*.

*GalE*-like ORFs were present in all the inserts sequenced, and in two cases, a *galE*-like ORF was co-localised with a transposase, suggesting it had recently been or could potentially be mobilised. This increases the likelihood that it may be a novel, environmental resistance mechanism, which could become clinically problematic if mobilised onto a promiscuous plasmid, as seen previously with *bla*_CTX−M_ (Cantón et al., 2012). This is especially concerning given *galE* genes have been shown to increase virulence, e.g. in extended spectrum β-lactamase -producing *Klebsiella pneumoniae* (Wang et al., 2020). Potentially mobilizable, novel β-lactamase encoding genes were identified from the same libraries in a previous study (Zhang et al., 2019), suggesting mobilisation potential could be common in these environments. *GalE* genes that confer resistance to antimicrobials (antibiotic, antifungal, and biocides) and salt have now been isolated from soil environments (this study), the human oral microbiome, the pig gut microbiome and from human and animal pathogens (Carlson-Banning et al., 2013; Culligan et al., 2012; Fry et al., 2000; Nakao et al., 2006; Nayak et al., 2006; Tansirichaiya et al., 2017). This suggests these genes possibly perform an important, yet overlooked, cross-resistance function across all “One Health” compartment microbiomes.

Interestingly, no variants of the well-characterised QAC efflux pump *qacE* were identified in this study. *QacE* genes are usually found at relatively high quantities in the environment, including previous research that studied the same environments used to generate these libraries (Gaze et al., 2005; Gaze et al., 2011). This further demonstrates the necessity of functional metagenomic studies to identify novel resistance determinants, that can even confer higher levels of resistance than known genes. For example, the BKC MIC of clones in this study was more than double (>84 μg/ml) of that conferred by the *qacE* gene (32 μg/ml).

In conclusion, for the first time, to our knowledge, we identified *galE* -like genes that confer cross-resistance to QACs and reduced susceptibility to antibiotics. *GalE*-like genes were the most common ORFs identified from these libraries, were associated with mobile elements, and conferred greater resistance to QACs than well-known QAC-resistance genes such as *qacE*. Our study and previous studies have now isolated *galE*-like genes from several different environments and bacterial species, suggesting this gene clade and potentially other genes in the LeLoir pathway could confer resistance to a range of antimicrobials and other abiotic environmental stressors in many environmental contexts. Further study into AMR conferred by mutated metabolic genes, including those in the LeLoir pathway, is warranted.

### Data access statement

The research data supporting this publication are provided within this paper and the supplementary information accompanying this publication. The sequence data are openly available from the University of Exeter's institutional repository Open Research Exeter (ORE) at https://doi.org/10.24378/exe.4524.

## CRediT authorship contribution statement

**Aimee K. Murray:** Formal analysis, Investigation, Methodology, Validation, Writing – original draft, Writing – review & editing, Visualization. **Lihong Zhang:** Investigation, Methodology, Writing – review & editing, Supervision, Conceptualization, Resources. **Jason Snape:** Writing – review & editing, Supervision, Project administration, Funding acquisition. **William H. Gaze:** Writing – review & editing, Supervision, Project administration, Funding acquisition, Conceptualization, Resources.

## Declaration of Competing Interest

The authors declare the following financial interests/personal relationships which may be considered as potential competing interests:

Jason Snape reports a relationship with AstraZeneca PLC that includes: employment and equity or stocks.

## Data Availability

Data will be made available on request.

## Appendix Supplementary materials

Image, application 1
