# Supplementary material for: Functional metagenomic libraries generated from anthropogenically impacted environments reveal importance of metabolic genes in biocide and antibiotic resistance
Source: Curr Res Microb Sci. 2023 Feb 26;4:100184. doi: 10.1016/j.crmicr.2023.100184 (PMC9995290; doi:10.1016/j.crmicr.2023.100184)
Supplement: Supplementary file 1 [file mmc1.docx]

Table S1. Calculation of coverage of the functional libraries, which equals the number of clones multiplied by the average insert size of the positive clones (30 clones tested) multiplied by the percentage of positive clones.

|  | Reed bed soil | Sewage cake | Grass Land soil |
| --- | --- | --- | --- |
| Numbers of clones | 210 | 386 | 630 |
| Average insert size (Kb) | 4.64 | 4.12 | 2.85 |
| Positive clone percentage | 65 | 65 | 75 |
| Coverage (Gb) | 0.63 | 1.03 | 1.53 |

Table S2. All the ORFs identified by primer walking all unique BKC resistant inserts. Shown is the name of the predicted protein, the % identity to the entry in the GenBank database, and the accession number for this highest hit. For some ORFs, multiple predicted proteins are shown with coverage in brackets. ORFs of particular interest (antimicrobial resistance genes or genes that logically could have roles in conferring resistance) are marked by ‘***’.

| Insert | ORF | % ID | Accession |
| --- | --- | --- | --- |
| GBKC *** | [UDP-glucose 4-epimerase [Chlorogloeopsis fritschii]](http://blast.ncbi.nlm.nih.gov/Blast.cgi#alnHdr_515379837) | 61 | [WP_016875182.1](http://www.ncbi.nlm.nih.gov/protein/515379837?report=genbank&log$=prottop&blast_rank=1&RID=J7NAZ2EU013) |
| *** | [UDP galactose 4-epimerase (EC 1.7.7.12) [Streptomyces lividans]](http://blast.ncbi.nlm.nih.gov/Blast.cgi#alnHdr_153261) | 52 | [AAA26747.1](http://www.ncbi.nlm.nih.gov/protein/153261?report=genbank&log$=prottop&blast_rank=1&RID=UYNZUJ7F016) |
| *** | [pyridoxal-dependent decarboxylase [Candidatus Koribacter versatilis]](http://blast.ncbi.nlm.nih.gov/Blast.cgi#alnHdr_499841352) | 54 | [WP_011522086.1](http://www.ncbi.nlm.nih.gov/protein/499841352?report=genbank&log$=prottop&blast_rank=1&RID=UYP5KWNZ013) |
| *** | [pyridoxal-dependent decarboxylase [Candidatus Koribacter versatilis]](http://blast.ncbi.nlm.nih.gov/Blast.cgi#alnHdr_499841352) | 70 | [WP_011522086.1](http://www.ncbi.nlm.nih.gov/protein/499841352?report=genbank&log$=prottop&blast_rank=1&RID=J7NBC5FP013) |
|  | [permease [Acetobacterium dehalogenans]](http://blast.ncbi.nlm.nih.gov/Blast.cgi#alnHdr_737374417) | 38 | [WP_035356200.1](http://www.ncbi.nlm.nih.gov/protein/737374417?report=genbank&log$=prottop&blast_rank=1&RID=UYP7DNT0013) |
|  | sulfur reduction protein DsrE [Dehalococcoidia bacterium SCGC AB-539-J10] | 28 | [WP_029475985.1](http://www.ncbi.nlm.nih.gov/protein/657673220?report=genbank&log$=prottop&blast_rank=1&RID=CSFE10KN01R) |
|  | [hypothetical protein [Burkholderia gladioli]](http://blast.ncbi.nlm.nih.gov/Blast.cgi#alnHdr_817522641) | 32 | [WP_046578597.1](http://www.ncbi.nlm.nih.gov/protein/817522641?report=genbank&log$=prottop&blast_rank=1&RID=CSFEC7TY01R) |
|  | [hypothetical protein [Kitasatospora phosalacinea]](http://blast.ncbi.nlm.nih.gov/Blast.cgi#alnHdr_702755308) | 41 | [WP_033256445.1](http://www.ncbi.nlm.nih.gov/protein/702755308?report=genbank&log$=prottop&blast_rank=1&RID=CSFFFKHX01R) |
|  | [hypothetical protein [Streptomyces sp. MspMP-M5]](http://blast.ncbi.nlm.nih.gov/Blast.cgi#alnHdr_517361372) | 39 | [WP_018536861.1](http://www.ncbi.nlm.nih.gov/protein/517361372?report=genbank&log$=prottop&blast_rank=1&RID=CSFJX32Z01R) |
|  |  |  |  |
| R2 *** | [peptide ABC transporter substrate-binding protein [Pseudomonas veronii]](http://blast.ncbi.nlm.nih.gov/Blast.cgi#alnHdr_817115849) | 96 | [WP_046488955.1](http://www.ncbi.nlm.nih.gov/protein/817115849?report=genbank&log$=prottop&blast_rank=1&RID=ZD8DYGMA01R) |
|  | [probable polyvinylalcohol dehydrogenase [Gimesia maris]](http://blast.ncbi.nlm.nih.gov/Blast.cgi#alnHdr_488726048) | 48 | [WP_002649604.1](http://www.ncbi.nlm.nih.gov/protein/488726048?report=genbank&log$=prottop&blast_rank=1&RID=ZD8E7HRY01R) |
|  | [nitrilase [Candidatus Solibacter usitatus]](http://blast.ncbi.nlm.nih.gov/Blast.cgi#alnHdr_500003418) | 77 | [WP_011684136.1](http://www.ncbi.nlm.nih.gov/protein/500003418?report=genbank&log$=prottop&blast_rank=1&RID=ZD8NNJS201R) |
|  | [hypothetical protein [Phyllobacterium sp. UNC302MFCol5.2]](http://blast.ncbi.nlm.nih.gov/Blast.cgi#alnHdr_652978942) with Domiain of Unknown Function (DUF) | 42 | [WP_027231546.1](http://www.ncbi.nlm.nih.gov/protein/652978942?report=genbank&log$=prottop&blast_rank=1&RID=ZD8R64BS01R) |
|  | [transcriptional regulator [Pseudomonas veronii]](http://blast.ncbi.nlm.nih.gov/Blast.cgi#alnHdr_814582433) | 100 | [WP_046381648.1](http://www.ncbi.nlm.nih.gov/protein/814582433?report=genbank&log$=prottop&blast_rank=1&RID=ZD8UJTCG01R) |
| *** | [peptide ABC transporter substrate-binding protein [Pseudomonas veronii]](http://blast.ncbi.nlm.nih.gov/Blast.cgi#alnHdr_814582432) | 84 | [WP_046381647.1](http://www.ncbi.nlm.nih.gov/protein/814582432?report=genbank&log$=prottop&blast_rank=1&RID=ZD8V72WV01R) |
| *** | [peptide ABC transporter substrate-binding protein [Pseudomonas veronii]](http://blast.ncbi.nlm.nih.gov/Blast.cgi#alnHdr_516457210) | 88 | [WP_017846050.1](http://www.ncbi.nlm.nih.gov/protein/516457210?report=genbank&log$=prottop&blast_rank=1&RID=ZD8VG23D01R) |
| *** | [UDP-glucose 4-epimerase [Planctopirus limnophila]](http://blast.ncbi.nlm.nih.gov/Blast.cgi#alnHdr_502874632) | 57 | [WP_013109608.1](http://www.ncbi.nlm.nih.gov/protein/502874632?report=genbank&log$=prottop&blast_rank=1&RID=ZD90XGNZ01R) |
|  | [aldehyde dehydrogenase [Pseudomonas veronii]](http://blast.ncbi.nlm.nih.gov/Blast.cgi#alnHdr_814582434) | 100 | [WP_046381649.1](http://www.ncbi.nlm.nih.gov/protein/814582434?report=genbank&log$=prottop&blast_rank=1&RID=ZD934BYJ01R) |
|  | [MULTISPECIES: 50S ribosomal protein L28 [Pseudomonas]](http://blast.ncbi.nlm.nih.gov/Blast.cgi#alnHdr_489269153) | 100 | [WP_003176907.1](http://www.ncbi.nlm.nih.gov/protein/489269153?report=genbank&log$=prottop&blast_rank=1&RID=ZD93G9PN01R) |
| *** | [UDP-glucose 4-epimerase [Planctopirus limnophila]](http://blast.ncbi.nlm.nih.gov/Blast.cgi#alnHdr_502874632) | 68 | [WP_013109608.1](http://www.ncbi.nlm.nih.gov/protein/502874632?report=genbank&log$=prottop&blast_rank=1&RID=ZD94CU8B01R) |
|  |  |  |  |
| R3 | [divalent ion tolerance protein CutA [Azoarcus sp. BH72]](http://blast.ncbi.nlm.nih.gov/Blast.cgi#alnHdr_500088044) | 73 | [WP_011764057.1](http://www.ncbi.nlm.nih.gov/protein/500088044?report=genbank&log$=prottop&blast_rank=1&RID=UYPMGANR013) |
| *** | [UDP-galactose 4-epimerase [uncultured prokaryote]](http://blast.ncbi.nlm.nih.gov/Blast.cgi#alnHdr_374854302) | 72 | [BAL57187.1](http://www.ncbi.nlm.nih.gov/protein/374854302?report=genbank&log$=prottop&blast_rank=1&RID=UYPS1JNR013) |
|  | [2-hydroxyhepta-2,4-diene-1,7-dioate isomerase [Anaerolinea thermophila]](http://blast.ncbi.nlm.nih.gov/Blast.cgi#alnHdr_503325122) | 61 | [WP_013559783.1](http://www.ncbi.nlm.nih.gov/protein/503325122?report=genbank&log$=prottop&blast_rank=1&RID=UYPSDAW7013) |
|  | [glutamyl-tRNA(Gln) amidotransferase [Anaerolinea thermophila]](http://blast.ncbi.nlm.nih.gov/Blast.cgi#alnHdr_503324087) | 75 | [WP_013558748.1](http://www.ncbi.nlm.nih.gov/protein/503324087?report=genbank&log$=prottop&blast_rank=1&RID=UYPU35ES013) |
|  | [2-hydroxyhepta-2,4-diene-1,7-dioate isomerase [Anaerolinea thermophila]](http://blast.ncbi.nlm.nih.gov/Blast.cgi#alnHdr_503325122) | 60 | [WP_013559783.1](http://www.ncbi.nlm.nih.gov/protein/503325122?report=genbank&log$=prottop&blast_rank=1&RID=UYPXN9N1013) |
|  | [thiol-disulfide interchange protein [Sulfuritalea hydrogenivorans sk43H]](http://blast.ncbi.nlm.nih.gov/Blast.cgi#alnHdr_572102818) | 72 | [BAO31229.1](http://www.ncbi.nlm.nih.gov/protein/572102818?report=genbank&log$=prottop&blast_rank=1&RID=T4P1MGFA015) |
|  | [riboflavin synthase subunit alpha [Sulfuritalea hydrogenivorans]](http://blast.ncbi.nlm.nih.gov/Blast.cgi#alnHdr_751633208) | 85 | [WP_041101012.1](http://www.ncbi.nlm.nih.gov/protein/751633208?report=genbank&log$=prottop&blast_rank=1&RID=UYR215AB013) |
|  | [chemotaxis protein [Massilia sp. LC238]](http://blast.ncbi.nlm.nih.gov/Blast.cgi#alnHdr_738260664) | 44 | [WP_036215602.1](http://www.ncbi.nlm.nih.gov/protein/738260664?report=genbank&log$=prottop&blast_rank=1&RID=UYR2A521016) |
| ***FIST DOMAIN | [hypothetical protein AZKH_2025 [Azoarcus sp. KH32C]](http://blast.ncbi.nlm.nih.gov/Blast.cgi#alnHdr_358637041) | 52 | [BAL24338.1](http://www.ncbi.nlm.nih.gov/protein/358637041?report=genbank&log$=prottop&blast_rank=1&RID=UYR8Z5AC013) |
|  | [Uncharacterised protein [Bordetella pertussis]](http://blast.ncbi.nlm.nih.gov/Blast.cgi#alnHdr_801362136) | 56 | [CFD85428.1](http://www.ncbi.nlm.nih.gov/protein/801362136?report=genbank&log$=prottop&blast_rank=1&RID=UYT8P0ZH016) |
|  | [PREDICTED: probable ribonuclease ZC3H12D isoform X3 [Macaca nemestrina]](http://blast.ncbi.nlm.nih.gov/Blast.cgi#alnHdr_795384698) | 43 | [XP_011751866.1](http://www.ncbi.nlm.nih.gov/protein/795384698?report=genbank&log$=prottop&blast_rank=1&RID=UYT9120F016) |
| *** | [hypothetical protein AW09_003777 [Candidatus Accumulibacter sp. BA-91]](http://blast.ncbi.nlm.nih.gov/Blast.cgi#alnHdr_668676377)  [penicillin-binding protein [Rhodococcus sp. ARP2]](http://blast.ncbi.nlm.nih.gov/Blast.cgi#alnHdr_837889387) | 62  30 | [KFB71096.1](http://www.ncbi.nlm.nih.gov/protein/668676377?report=genbank&log$=prottop&blast_rank=1&RID=UYTFYDNV016)  [WP_047889119.1](http://www.ncbi.nlm.nih.gov/protein/837889387?report=genbank&log$=prottop&blast_rank=6&RID=UYTFYDNV016) |
|  | [DUF1745 domain-containing protein [Methylobacter tundripaludum]](http://blast.ncbi.nlm.nih.gov/Blast.cgi#alnHdr_493949743) | 65 | [WP_006893638.1](http://www.ncbi.nlm.nih.gov/protein/493949743?report=genbank&log$=prottop&blast_rank=1&RID=UYT6Y62J013) |
|  |  |  |  |
| R10 | [glycerol kinase [Perlucidibaca piscinae]](http://blast.ncbi.nlm.nih.gov/Blast.cgi#alnHdr_551337271) | 77 | [WP_022956698.1](http://www.ncbi.nlm.nih.gov/protein/551337271?report=genbank&log$=prottop&blast_rank=1&RID=UW3N32WE01N) |
|  | [hypothetical protein [Citromicrobium bathyomarinum]](http://blast.ncbi.nlm.nih.gov/Blast.cgi#alnHdr_750073878) | 47 | [WP_040378562.1](http://www.ncbi.nlm.nih.gov/protein/750073878?report=genbank&log$=prottop&blast_rank=1&RID=UW3NED1B01N) |
| *** | [UDP-galactose-4-epimerase [Bacillus selenatarsenatis]](http://blast.ncbi.nlm.nih.gov/Blast.cgi#alnHdr_754570661) | 72 | [WP_041963976.1](http://www.ncbi.nlm.nih.gov/protein/754570661?report=genbank&log$=prottop&blast_rank=1&RID=UW3NUWZW01N) |
|  | [UDP-glucose 6-dehydrogenase [Acidovorax delafieldii]](http://blast.ncbi.nlm.nih.gov/Blast.cgi#alnHdr_492267757) | 28 | [WP_005794330.1](http://www.ncbi.nlm.nih.gov/protein/492267757?report=genbank&log$=prottop&blast_rank=1&RID=UW3RGDTU01N) |
| *** | [hypothetical protein [Bacillus thuringiensis]](http://blast.ncbi.nlm.nih.gov/Blast.cgi#alnHdr_503320411)  [transposase [Escherichia coli]](http://blast.ncbi.nlm.nih.gov/Blast.cgi#alnHdr_502333355)  [beta galactosidase alpha [Cloning vector pNOT218]](http://blast.ncbi.nlm.nih.gov/Blast.cgi#alnHdr_18766963) | 62  68  67 | [WP_013555072.1](http://www.ncbi.nlm.nih.gov/protein/503320411?report=genbank&log$=prottop&blast_rank=1&RID=UW3S1BW301N)  [WP_012766388.1](http://www.ncbi.nlm.nih.gov/protein/502333355?report=genbank&log$=prottop&blast_rank=2&RID=UW3S1BW301N)  [AAL79196.1](http://www.ncbi.nlm.nih.gov/protein/18766963?report=genbank&log$=prottop&blast_rank=5&RID=UW3S1BW301N) |
| *** | [UDP-galactose-4-epimerase [Rhodopirellula sallentina]](http://blast.ncbi.nlm.nih.gov/Blast.cgi#alnHdr_763474252) | 68 | [WP_044303185.1](http://www.ncbi.nlm.nih.gov/protein/763474252?report=genbank&log$=prottop&blast_rank=1&RID=UW41CGPA01N) |
|  | [hypothetical protein [Balneatrix alpica]](http://blast.ncbi.nlm.nih.gov/Blast.cgi#alnHdr_653061370) | 52 | [WP_027312693.1](http://www.ncbi.nlm.nih.gov/protein/653061370?report=genbank&log$=prottop&blast_rank=1&RID=UW41TMV501N) |
|  | [glucose-6-phosphate isomerase [Psychrobacter cryohalolentis]](http://blast.ncbi.nlm.nih.gov/Blast.cgi#alnHdr_499831759) | 60 | [WP_011512493.1](http://www.ncbi.nlm.nih.gov/protein/499831759?report=genbank&log$=prottop&blast_rank=1&RID=UW45RF6T01N) |
| *** | [ABC transporter permease [Streptomyces galbus]](http://blast.ncbi.nlm.nih.gov/Blast.cgi#alnHdr_716909113) | 63 | [WP_033524975.1](http://www.ncbi.nlm.nih.gov/protein/716909113?report=genbank&log$=prottop&blast_rank=1&RID=UW4992C801N) |
|  |  |  |  |
| R14  *** | [hypothetical protein [Firmicutes bacterium CAG:646]](http://blast.ncbi.nlm.nih.gov/Blast.cgi#alnHdr_547276759)  [UDP-galactose-4-epimerase [Dasania marina]](http://blast.ncbi.nlm.nih.gov/Blast.cgi#alnHdr_518360114) | 70  71 | [WP_022010496.1](http://www.ncbi.nlm.nih.gov/protein/547276759?report=genbank&log$=prottop&blast_rank=1&RID=UJ53N16T014)  [WP_019530321.1](http://www.ncbi.nlm.nih.gov/protein/518360114?report=genbank&log$=prottop&blast_rank=2&RID=UJ53N16T014) |
|  | [putative polysaccharide transport system component signal peptide protein (ragA) [Ralstonia solanacearum PSI07]](http://blast.ncbi.nlm.nih.gov/Blast.cgi#alnHdr_299077872) | 42 | [CBJ50510.1](http://www.ncbi.nlm.nih.gov/protein/299077872?report=genbank&log$=prottop&blast_rank=2&RID=UJ54WFVY014) |
|  |  |  |  |
| R17 | [hypothetical protein [Nocardioides insulae]](http://blast.ncbi.nlm.nih.gov/Blast.cgi#alnHdr_655249344) | 57 | [WP_028660028.1](http://www.ncbi.nlm.nih.gov/protein/655249344?report=genbank&log$=prottop&blast_rank=1&RID=XT12DS1N014) |
|  | [hypothetical protein [Burkholderiales bacterium GJ-E10]](http://blast.ncbi.nlm.nih.gov/Blast.cgi#alnHdr_780094115) | 60 | [WP_045469855.1](http://www.ncbi.nlm.nih.gov/protein/780094115?report=genbank&log$=prottop&blast_rank=1&RID=XT12RNVK014) |
|  | [hypothetical protein [Rubrivivax gelatinosus]](http://blast.ncbi.nlm.nih.gov/Blast.cgi#alnHdr_916247554) | 40 | [WP_050985547.1](http://www.ncbi.nlm.nih.gov/protein/916247554?report=genbank&log$=prottop&blast_rank=1&RID=XT14PU5C014) |
|  | [hypothetical protein [Aquincola tertiaricarbonis]](http://blast.ncbi.nlm.nih.gov/Blast.cgi#alnHdr_805543603) | 47 | [WP_046115559.1](http://www.ncbi.nlm.nih.gov/protein/805543603?report=genbank&log$=prottop&blast_rank=1&RID=XT14BR2G015) |
| *** | [conserved hypothetical protein [Novosphingobium sp. KN65.2]](http://blast.ncbi.nlm.nih.gov/Blast.cgi#alnHdr_808403671) | 52 | [CDO35116.1](http://www.ncbi.nlm.nih.gov/protein/808403671?report=genbank&log$=prottop&blast_rank=1&RID=XT1J6VB9015) |
|  | [Ankyrin repeat and death domain-containing protein 1A [Pteropus alecto]](http://blast.ncbi.nlm.nih.gov/Blast.cgi#alnHdr_431895914) | 51 | [ELK05332.1](http://www.ncbi.nlm.nih.gov/protein/431895914?report=genbank&log$=prottop&blast_rank=1&RID=XT1TAS7B015) |
| *** | [hypothetical protein [Aphanizomenon flos-aquae]](http://blast.ncbi.nlm.nih.gov/Blast.cgi#alnHdr_653153379)  [UDP-galactopyranose mutase [Haliscomenobacter hydrossis]](http://blast.ncbi.nlm.nih.gov/Blast.cgi#alnHdr_503531721) | 64/65  51/62 | [WP_027402397.1](http://www.ncbi.nlm.nih.gov/protein/653153379?report=genbank&log$=prottop&blast_rank=1&RID=XT1WD41X015)  [WP_013765800.1](http://www.ncbi.nlm.nih.gov/protein/503531721?report=genbank&log$=prottop&blast_rank=4&RID=XT1WD41X015)/  [WP_013765800.1](http://www.ncbi.nlm.nih.gov/protein/503531721?report=genbank&log$=prottop&blast_rank=4&RID=XT1YW5TM015) |
| *** | [MULTISPECIES: membrane protein [Streptomyces]](http://blast.ncbi.nlm.nih.gov/Blast.cgi#alnHdr_739807240) | 55 | [WP_037659172.1](http://www.ncbi.nlm.nih.gov/protein/739807240?report=genbank&log$=prottop&blast_rank=1&RID=XT1ZER9B014) |
|  |  |  |  |
| R24 *** | [UDP-glucose 4-epimerase [Pseudomonas sp. 11/12A]](http://blast.ncbi.nlm.nih.gov/Blast.cgi#alnHdr_835504333) | 69 | [WP_047530443.1](http://www.ncbi.nlm.nih.gov/protein/835504333?report=genbank&log$=prottop&blast_rank=1&RID=ACDUG70F014) |
|  | [aminoglycoside phosphotransferase [Streptomyces aureofaciens]](http://blast.ncbi.nlm.nih.gov/Blast.cgi#alnHdr_919504155) | 33 | [WP_052838580.1](http://www.ncbi.nlm.nih.gov/protein/919504155?report=genbank&log$=prottop&blast_rank=1&RID=ACDUYGJ2014) |
|  | [protein tyrosine kinase [Pseudomonas sp. 11/12A]](http://blast.ncbi.nlm.nih.gov/Blast.cgi#alnHdr_835504336) | 92 | [WP_047530445.1](http://www.ncbi.nlm.nih.gov/protein/835504336?report=genbank&log$=prottop&blast_rank=1&RID=ACDVDZGC014) |
|  | [hypothetical protein [Pseudomonas sp. 11/12A]](http://blast.ncbi.nlm.nih.gov/Blast.cgi#alnHdr_835504345) | 98 | [WP_047530451.1](http://www.ncbi.nlm.nih.gov/protein/835504345?report=genbank&log$=prottop&blast_rank=1&RID=ACDVWBY1014) |
| *** | [sulfatase [Pseudomonas sp. 11/12A]](http://blast.ncbi.nlm.nih.gov/Blast.cgi#alnHdr_835504329) | 88 | [WP_047530441.1](http://www.ncbi.nlm.nih.gov/protein/835504329?report=genbank&log$=prottop&blast_rank=1&RID=ACDWAMPC015) |
|  | [protein tyrosine kinase [Pseudomonas sp. 11/12A]](http://blast.ncbi.nlm.nih.gov/Blast.cgi#alnHdr_835504336) | 91 | [WP_047530445.1](http://www.ncbi.nlm.nih.gov/protein/835504336?report=genbank&log$=prottop&blast_rank=1&RID=ACDWRB39014) |
| *** | [lipopolysaccharide biosynthesis protein [Pseudomonas sp. 11/12A]](http://blast.ncbi.nlm.nih.gov/Blast.cgi#alnHdr_835504339) | 99 | [lipopolysaccharide biosynthesis protein [Pseudomonas sp. 11/12A]](http://blast.ncbi.nlm.nih.gov/Blast.cgi#alnHdr_835504339) |
| *** | [sugar ABC transporter substrate-binding protein [Pseudomonas sp. 11/12A]](http://blast.ncbi.nlm.nih.gov/Blast.cgi#alnHdr_835504349) | 97 | [WP_047530453.1](http://www.ncbi.nlm.nih.gov/protein/835504349?report=genbank&log$=prottop&blast_rank=1&RID=ACE8AV9W014) |
| *** | [sulfatase [Pseudomonas sp. 11/12A]](http://blast.ncbi.nlm.nih.gov/Blast.cgi#alnHdr_835504329) | 96 | [WP_047530441.1](http://www.ncbi.nlm.nih.gov/protein/835504329?report=genbank&log$=prottop&blast_rank=1&RID=ACE8PM0E014) |
|  |  |  |  |
| S2 *** | [MFS transporter family protein [Candidatus Cloacimonas acidaminovorans]](http://blast.ncbi.nlm.nih.gov/Blast.cgi#alnHdr_505238141) | 36 | [WP_015425243.1](http://www.ncbi.nlm.nih.gov/protein/505238141?report=genbank&log$=prottop&blast_rank=1&RID=UYU9D34D013) |
| *** | [UDP-glucose 4-epimerase [Anaerolinea thermophila]](http://blast.ncbi.nlm.nih.gov/Blast.cgi#alnHdr_503326666) | 74 | [WP_013561327.1](http://www.ncbi.nlm.nih.gov/protein/503326666?report=genbank&log$=prottop&blast_rank=1&RID=UYU9MS7E016) |
|  | [hypothetical protein [bacterium JGI-5]](http://blast.ncbi.nlm.nih.gov/Blast.cgi#alnHdr_825282655)  [protease [Peptococcaceae bacterium BICA1-7]](http://blast.ncbi.nlm.nih.gov/Blast.cgi#alnHdr_734874356) | 58  56 | [WP_047133258.1](http://www.ncbi.nlm.nih.gov/protein/825282655?report=genbank&log$=prottop&blast_rank=1&RID=UYUC15XV016)  [WP_034123852.1](http://www.ncbi.nlm.nih.gov/protein/734874356?report=genbank&log$=prottop&blast_rank=3&RID=UYUC15XV016) |
|  |  |  |  |
| S3 *** | [oxidoreductase [Hassallia byssoidea]](http://blast.ncbi.nlm.nih.gov/Blast.cgi#alnHdr_748172481) | 67 | [WP_039746039.1](http://www.ncbi.nlm.nih.gov/protein/748172481?report=genbank&log$=prottop&blast_rank=1&RID=UHZGFVBJ015) |
| *** | [UDP-galactose-4-epimerase [Draconibacterium orientale]](http://blast.ncbi.nlm.nih.gov/Blast.cgi#alnHdr_740772840) | 66 | [WP_038558124.1](http://www.ncbi.nlm.nih.gov/protein/740772840?report=genbank&log$=prottop&blast_rank=1&RID=UHZH8VA4015) |
|  | [dTDP-glucose 4,6-dehydratase [Thermophagus xiamenensis]](http://blast.ncbi.nlm.nih.gov/Blast.cgi#alnHdr_498212472) | 70 | [WP_010526628.1](http://www.ncbi.nlm.nih.gov/protein/498212472?report=genbank&log$=prottop&blast_rank=1&RID=UHZKASBY014) |
|  | [UDP-N-acetyl-D-galactosamine dehydrogenase [Adhaeribacter aquaticus]](http://blast.ncbi.nlm.nih.gov/Blast.cgi#alnHdr_651339237) | 68 | [WP_026462223.1](http://www.ncbi.nlm.nih.gov/protein/651339237?report=genbank&log$=prottop&blast_rank=1&RID=UHZRY7RW014) |
|  |  |  |  |
| S4 | [hypothetical protein [Escherichia coli]](http://blast.ncbi.nlm.nih.gov/Blast.cgi#alnHdr_919936566) | 87 | [WP_052913615.1](http://www.ncbi.nlm.nih.gov/protein/919936566?report=genbank&log$=prottop&blast_rank=1&RID=XSU6JXXH01R) |
| ***  *** | [hypothetical protein UUU_05190 [Klebsiella pneumoniae subsp. pneumoniae DSM 30104]](http://blast.ncbi.nlm.nih.gov/Blast.cgi#alnHdr_397745326)  [Multidrug resistance protein mdtB [Erwinia amylovora MR1]](http://blast.ncbi.nlm.nih.gov/Blast.cgi#alnHdr_478729573)  [membrane protein [Thiorhodococcus sp. AK35]](http://blast.ncbi.nlm.nih.gov/Blast.cgi#alnHdr_760073043) | 58  42  49 | [EJK92533.1](http://www.ncbi.nlm.nih.gov/protein/397745326?report=genbank&log$=prottop&blast_rank=1&RID=XSU6YJTC01R)  [CCP06064.1](http://www.ncbi.nlm.nih.gov/protein/478729573?report=genbank&log$=prottop&blast_rank=3&RID=XSU6YJTC01R)  [WP_043755805.1](http://www.ncbi.nlm.nih.gov/protein/760073043?report=genbank&log$=prottop&blast_rank=4&RID=XSU6YJTC01R) |
|  | [FIG00732864: hypothetical protein [Klebsiella pneumoniae IS10]](http://blast.ncbi.nlm.nih.gov/Blast.cgi#alnHdr_571202862) | 55 | [CDK62066.1](http://www.ncbi.nlm.nih.gov/protein/571202862?report=genbank&log$=prottop&blast_rank=1&RID=XSU77ZJP01R) |
|  | [hypothetical protein WRSd5_03586 [Shigella dysenteriae WRSd5]](http://blast.ncbi.nlm.nih.gov/Blast.cgi#alnHdr_559657378) | 95 | [ESU79657.1](http://www.ncbi.nlm.nih.gov/protein/559657378?report=genbank&log$=prottop&blast_rank=1&RID=XSU7HF2P01R) |
| *** | [tetracycline resistance protein, class A [Escherichia coli BWH 24]](http://blast.ncbi.nlm.nih.gov/Blast.cgi#alnHdr_550203129) | 93 | [ERO93707.1](http://www.ncbi.nlm.nih.gov/protein/550203129?report=genbank&log$=prottop&blast_rank=1&RID=XSU8Z6J001R) |
|  | [hypothetical protein [Salmonella enterica]](http://blast.ncbi.nlm.nih.gov/Blast.cgi#alnHdr_485745768) | 100 | [WP_001372230.1](http://www.ncbi.nlm.nih.gov/protein/485745768?report=genbank&log$=prottop&blast_rank=1&RID=XSUFRF2J01R) |
|  | [hypothetical protein HMPREF9551_04418 [Escherichia coli MS 196-1]](http://blast.ncbi.nlm.nih.gov/Blast.cgi#alnHdr_299878416) | 100 | [EFI86627.1](http://www.ncbi.nlm.nih.gov/protein/299878416?report=genbank&log$=prottop&blast_rank=1&RID=XSUMCDC101R) |
| *** | [UDP-glucose 4-epimerase [Anaerolinea thermophila]](http://blast.ncbi.nlm.nih.gov/Blast.cgi#alnHdr_503326666) | 68 | [WP_013561327.1](http://www.ncbi.nlm.nih.gov/protein/503326666?report=genbank&log$=prottop&blast_rank=1&RID=XSUN1GBT01R) |
| *** | [MULTISPECIES: MFS transporter [unclassified Cloacimonetes]](http://blast.ncbi.nlm.nih.gov/Blast.cgi#alnHdr_661254376) | 42 | [WP_029949877.1](http://www.ncbi.nlm.nih.gov/protein/661254376?report=genbank&log$=prottop&blast_rank=1&RID=XSUW9K6501R) |
| *** | [UDP-galactose 4-epimerase [Leptolinea tardivitalis]](http://blast.ncbi.nlm.nih.gov/Blast.cgi#alnHdr_913166200) | 76 | [GAP20355.1](http://www.ncbi.nlm.nih.gov/protein/913166200?report=genbank&log$=prottop&blast_rank=1&RID=XSUXM6JW01R) |
|  | [lactose operon repressor domain protein [Staphylococcus aureus Lyso 2 2010]](http://blast.ncbi.nlm.nih.gov/Blast.cgi#alnHdr_659929545) | 93 | [KEK30836.1](http://www.ncbi.nlm.nih.gov/protein/659929545?report=genbank&log$=prottop&blast_rank=1&RID=XSUYMJ9N01R) |
| *** | [tetracycline resistance MFS efflux pump [Escherichia coli]](http://blast.ncbi.nlm.nih.gov/Blast.cgi#alnHdr_499491969) | 100 | [WP_011178609.1](http://www.ncbi.nlm.nih.gov/protein/499491969?report=genbank&log$=prottop&blast_rank=1&RID=XSUYARG501R) |
| *** | [putative transposase [Escherichia coli 3-020-07_S4_C1]](http://blast.ncbi.nlm.nih.gov/Blast.cgi#alnHdr_658721096) | 100 (42% cover) | [KEJ62634.1](http://www.ncbi.nlm.nih.gov/protein/658721096?report=genbank&log$=prottop&blast_rank=1&RID=XSV8TCK901R) |
|  | [transcriptional regulator AraC [Broad host range vector pMLBAD]](http://blast.ncbi.nlm.nih.gov/Blast.cgi#alnHdr_22074790) | 99 | [AAM63382.1](http://www.ncbi.nlm.nih.gov/protein/22074790?report=genbank&log$=prottop&blast_rank=1&RID=XSVA0WBJ01R) |
|  | [MULTISPECIES: hypothetical protein [Enterobacteriaceae]](http://blast.ncbi.nlm.nih.gov/Blast.cgi#alnHdr_445950353) | 100 (86% cover) | [WP_000028208.1](http://www.ncbi.nlm.nih.gov/protein/445950353?report=genbank&log$=prottop&blast_rank=1&RID=XSZG2WSS015) |
|  | [hypothetical protein [Escherichia coli]](http://blast.ncbi.nlm.nih.gov/Blast.cgi#alnHdr_501804914) | 96 (44% cover) | [WP_012644004.1](http://www.ncbi.nlm.nih.gov/protein/501804914?report=genbank&log$=prottop&blast_rank=1&RID=XSZGCPXR015) |
| *** | [Transcriptional regulator AraC [Shigella sonnei]](http://blast.ncbi.nlm.nih.gov/Blast.cgi#alnHdr_903533106)  [zinc ABC transporter ATPase [Salmonella enterica subsp. enterica serovar Enteritidis str. EC20121178]](http://blast.ncbi.nlm.nih.gov/Blast.cgi#alnHdr_602767781) | 96  67 | CSP96607.1\|  [AHO12049.1](http://www.ncbi.nlm.nih.gov/protein/602767781?report=genbank&log$=prottop&blast_rank=2&RID=XSZGMBMK015) |
| *** | [uncharacterized membrane protein [Longilinea arvoryzae]](http://blast.ncbi.nlm.nih.gov/Blast.cgi#alnHdr_902955222) | 66 | [GAP15674.1](http://www.ncbi.nlm.nih.gov/protein/902955222?report=genbank&log$=prottop&blast_rank=1&RID=XSZH6FT6014) |
| *** | [tetracycline repressor protein class A transposon 1721 [Klebsiella pneumoniae]](http://blast.ncbi.nlm.nih.gov/Blast.cgi#alnHdr_839690843) | 95 | [KMH64585.1](http://www.ncbi.nlm.nih.gov/protein/839690843?report=genbank&log$=prottop&blast_rank=1&RID=XSZKMUY1015) |
|  | [hypothetical protein HPMG_01967 [Helicobacter pullorum MIT 98-5489]](http://blast.ncbi.nlm.nih.gov/Blast.cgi#alnHdr_239524644) | 60 | [EEQ64510.1](http://www.ncbi.nlm.nih.gov/protein/239524644?report=genbank&log$=prottop&blast_rank=1&RID=XSZHTAAU015) |
|  | [fumarate hydratase [delta proteobacterium MLMS-1]](http://blast.ncbi.nlm.nih.gov/Blast.cgi#alnHdr_494505713) | 50 | WP_007295173.1\| |
| *** | [tetracycline repressor protein class A [uncultured bacterium]](http://blast.ncbi.nlm.nih.gov/Blast.cgi#alnHdr_403398793) | 96 (80% cover) | [AFR44371.1](http://www.ncbi.nlm.nih.gov/protein/403398793?report=genbank&log$=prottop&blast_rank=1&RID=XSZV7KKP015) |
|  | [conserved hypothetical protein [Escherichia coli 042]](http://blast.ncbi.nlm.nih.gov/Blast.cgi#alnHdr_284923836) | 100 | CBG36934.1\| |
|  | [LacOPZ-alpha peptide from pUC9; putative [unidentified cloning vector]](http://blast.ncbi.nlm.nih.gov/Blast.cgi#alnHdr_994736) | 93 (84% cover) | [AAA75561.1](http://www.ncbi.nlm.nih.gov/protein/994736?report=genbank&log$=prottop&blast_rank=1&RID=XSZZ17JM015) |
|  | [hypothetical protein [Methanosarcina mazei]](http://blast.ncbi.nlm.nih.gov/Blast.cgi#alnHdr_850518515)  [pyruvate formate lyase-activating enzyme 1 [Escherichia coli KO11FL]](http://blast.ncbi.nlm.nih.gov/Blast.cgi#alnHdr_383393658) | 72 (96 cover)  74 (62 cover) | [WP_048049241.1](http://www.ncbi.nlm.nih.gov/protein/850518515?report=genbank&log$=prottop&blast_rank=1&RID=XT006DCF015)  [AFH18616.1](http://www.ncbi.nlm.nih.gov/protein/383393658?report=genbank&log$=prottop&blast_rank=2&RID=XT006DCF015) |
|  | [hypothetical protein [Achromobacter arsenitoxydans]](http://blast.ncbi.nlm.nih.gov/Blast.cgi#alnHdr_495441669)  [putative acetyltransferase YhhY [Salmonella enterica subsp. enterica serovar Typhimurium str. DT104]](http://blast.ncbi.nlm.nih.gov/Blast.cgi#alnHdr_898353482) | 100 (40% cover) | [WP_008166363.1](http://www.ncbi.nlm.nih.gov/protein/495441669?report=genbank&log$=prottop&blast_rank=1&RID=XT01F4XY015)  [CQA99509.1](http://www.ncbi.nlm.nih.gov/protein/898353482?report=genbank&log$=prottop&blast_rank=3&RID=XT01F4XY015) |
| *** | [UDP-glucose 4-epimerase [Anaerolinea thermophila]](http://blast.ncbi.nlm.nih.gov/Blast.cgi#alnHdr_503326666) | 68 | [WP_013561327.1](http://www.ncbi.nlm.nih.gov/protein/503326666?report=genbank&log$=prottop&blast_rank=1&RID=YXZEFPPG015) |
|  | [PREDICTED: E3 ubiquitin-protein ligase synoviolin-like [Nicotiana sylvestris]](http://blast.ncbi.nlm.nih.gov/Blast.cgi#alnHdr_698478724) | 34 | \|XP_009786500.1\| |
| *** | [MFS transporter family protein [Candidatus Cloacimonas acidaminovorans]](http://blast.ncbi.nlm.nih.gov/Blast.cgi#alnHdr_505238141) | 36 | [WP_015425243.1](http://www.ncbi.nlm.nih.gov/protein/505238141?report=genbank&log$=prottop&blast_rank=1&RID=YXZF2SPV015) |
| *** | [UDP-galactose 4-epimerase [Leptolinea tardivitalis]](http://blast.ncbi.nlm.nih.gov/Blast.cgi#alnHdr_913166200) | 76 | [GAP20355.1](http://www.ncbi.nlm.nih.gov/protein/913166200?report=genbank&log$=prottop&blast_rank=1&RID=YXZFNU4C014) |
| *** | [uncharacterized membrane protein [Longilinea arvoryzae]](http://blast.ncbi.nlm.nih.gov/Blast.cgi#alnHdr_902955222) | 66 | [GAP15674.1](http://www.ncbi.nlm.nih.gov/protein/902955222?report=genbank&log$=prottop&blast_rank=1&RID=YXZMJCRU014) |
|  | [titin2 [Bombyx mori]](http://blast.ncbi.nlm.nih.gov/Blast.cgi#alnHdr_148298772) | 36 | NP_001091843.1\| |
|  |  |  |  |
| S21 | [ferrochelatase [Photobacterium damselae]](http://blast.ncbi.nlm.nih.gov/Blast.cgi#alnHdr_738876146) | 48 | [WP_036764187.1](http://www.ncbi.nlm.nih.gov/protein/738876146?report=genbank&log$=prottop&blast_rank=1&RID=UHKSDZ5F014) |
|  | [UDP pyrophosphate phosphatase [Thalassolituus oleivorans]](http://blast.ncbi.nlm.nih.gov/Blast.cgi#alnHdr_643876441) | 63 | [WP_025266051.1](http://www.ncbi.nlm.nih.gov/protein/643876441?report=genbank&log$=prottop&blast_rank=1&RID=UHKSXEF2014) |
|  | [tyrosine protein kinase [Pseudomonas sp. GM49]](http://blast.ncbi.nlm.nih.gov/Blast.cgi#alnHdr_495274695) | 51 | [WP_007999450.1](http://www.ncbi.nlm.nih.gov/protein/495274695?report=genbank&log$=prottop&blast_rank=1&RID=UHKUGDBE015) |
|  | [phosphotyrosine protein phosphatase [Pseudomonas pelagia]](http://blast.ncbi.nlm.nih.gov/Blast.cgi#alnHdr_551344935) | 52 | [WP_022964347.1](http://www.ncbi.nlm.nih.gov/protein/551344935?report=genbank&log$=prottop&blast_rank=1&RID=UHKW39U3015) |
| *** | [MULTISPECIES: UDP-galactose-4-epimerase [Alcanivorax]](http://blast.ncbi.nlm.nih.gov/Blast.cgi#alnHdr_737478752) | 70 | [WP_035458552.1](http://www.ncbi.nlm.nih.gov/protein/737478752?report=genbank&log$=prottop&blast_rank=1&RID=UHKYX55Z014) |
|  | [hypothetical protein [Pseudomonas nitroreducens]](http://blast.ncbi.nlm.nih.gov/Blast.cgi#alnHdr_516090577) | 55 | [WP_017521157.1](http://www.ncbi.nlm.nih.gov/protein/516090577?report=genbank&log$=prottop&blast_rank=1&RID=UHM72VD6014) |
| *** | [capsular polysaccharide biosynthesis protein [Pseudomonas fluorescens]](http://blast.ncbi.nlm.nih.gov/Blast.cgi#alnHdr_489303911) | 53 (97 cover) | [WP_003211361.1](http://www.ncbi.nlm.nih.gov/protein/489303911?report=genbank&log$=prottop&blast_rank=1&RID=UHM8UJ3A015) |
| *** | [capsular polysaccharide biosynthesis protein [Pseudomonas sp. M1]](http://blast.ncbi.nlm.nih.gov/Blast.cgi#alnHdr_568075060) | 44 (67) | [ETM67913.1](http://www.ncbi.nlm.nih.gov/protein/568075060?report=genbank&log$=prottop&blast_rank=1&RID=UHMEXVP9014) |

Table S3. Table showing sequence similarity between UDP-galactose-4-epimerases identified in this study and in the study by [Kazimierczak et al. (2009)](#_ENREF_150) (‘Clone 9’ and ‘Clone 15’). M = Megablast, n = blastn. The first number is the sequence identity, the second number following the / is the percentage coverage. Alignment was performed with NCBI blastn for alignment of two sequences.

|  | R76 | R87 | R109 | R147 | R161 | R241 | S59 | S62 | S70 | S78 | S167 | S178 | S188 | S2-1 | Rb14-46 | Clone 9 | Clone 15 |
| --- | --- | --- | --- | --- | --- | --- | --- | --- | --- | --- | --- | --- | --- | --- | --- | --- | --- |
| R11 | 94N/3 | 99M | 66N/10 | 93N/2 | 66N/38 | 66N/10 | 90N/1 | 100N/1 | 100N/1 | 67N/10 | 93N/1 | 100N/1 | 67N/9 | 76N/2 | 68N/30 | NO ID | NO ID |
| R76 |  | 94N/2 | 98M | 93N/0 | 86N/2 | 99M | 100N/1 | 97N/8 | 100N/0 | 100N/1 | 100N/0 | 91N/9 | 100N/1 | 100N/0 | 86N/1 | 94N/4 | NO ID |
| R87 |  |  | 66N/10 | 93N/1 | 66N/44 | 66N/10 | 90N/2 | 100N/2 | 93N/2 | 67N/9 | 93N/2 | 100N/2 | 67N/9 | 76N/3 | 68N/31 | NO ID | NO ID |
| R109 |  |  |  | 100N/1 | 86N/1 | 98M | 100N/2 | 97N/7 | 100N/1 | 100N/1 | 100N/1 | 91N/7 | 100N/1 | 100N/0 | 86N/0 | 81N/7 | 74N/6 |
| R147 |  |  |  |  | 100N/2 | 100N/2 | 93N/3 | 100N/1 | 100N/1 | 93N/1 | 100N/1 | NO ID | 93N/1 | 100N/1 | 100N/2 | NO ID | 100N/1 |
| R161 |  |  |  |  |  | 86N/2 | NO ID | NO ID | NO ID | 100N/1 | NO ID | NO ID | 100N/0 | NO ID | 98M | NO ID | 70N/25 |
| R241 |  |  |  |  |  |  | 100N/2 | 97N/7 | NO ID | 100N/1 | NO ID | 91N/9 | 100N/1 | NO ID | 86N/1 | 80N/7 | NO ID |
| S59 |  |  |  |  |  |  |  | 100N/2 | 100N/1 | 79M | 100N/0 | 100N/2 | 78M | 100N/0 | NO ID | 81N/2 | 100N/1 |
| S62 |  |  |  |  |  |  |  |  | 98M | 94N/4 | 98M | 95M | 100N/1 | 98M | 100N/0 | 93N/1 | NO ID |
| S70 |  |  |  |  |  |  |  |  |  | 94N/2 | 99M | 95M | NO ID | 98M | 83/3 | 93N/1 | NO ID |
| S78 |  |  |  |  |  |  |  |  |  |  | 94N/2 | 94N/2 | 98M | 94N/2 | 100N/0 | 66N/55 | NO ID |
| S167 |  |  |  |  |  |  |  |  |  |  |  | 95M | 100N/0 | 98M | 93N/0 | 93N/1 | NO ID |
